# Probable progressive multifocal leukoencephalopathy-immune reconstitution inflammatory syndrome with immunosuppressant dose reduction following lung transplantation: a case report and literature review

**DOI:** 10.1186/s12883-019-1493-1

**Published:** 2019-10-31

**Authors:** Kazuhiro Ishii, Fumiko Yamamoto, Shinsuke Homma, Yoshinori Okada, Kazuo Nakamichi, Masayuki Saijo, Akira Tamaoka

**Affiliations:** 1 Department of Neurology, Division of Clinical Medicine, Faculty of Medicine, University of Tsukuba, 1-1-1 Ten’noudai, Tsukuba, Ibaraki, 305-8575 Japan; 2 Department of Pulmonology, Division of Clinical Medicine, Faculty of Medicine, University of Tsukuba, 1-1-1 Ten’noudai, Tsukuba, Ibaraki, 305-8575 Japan; 3 Department of Thoracic Surgery, Institute of Development, Aging and Cancer, Tohoku University, 4-1 Seiryomachi, Aoba-ku Sendai, 980-8575 Japan; 4 Department of Virology 1, National Institute of Infectious Diseases, Toyama 1-23-1, Shinjuku-ku, Tokyo 162-8640 Japan

**Keywords:** Progressive multifocal leukoencephalopathy, Mefloquine, CD4 positive cell, JC polyomavirus, Lung transplantation, Immune reconstitution inflammatory syndrome

## Abstract

**Background:**

Progressive multifocal leukoencephalopathy (PML) is a rapidly developing demyelinating disease in the cerebral white matter and is often caused by JC polyomavirus (JCV). PML after lung transplantation is rare and has a poor prognosis, with no established therapies. Reducing the patient’s immunosuppressant doses, thereby restoring immunity, could be used to treat PML. However, some patients develop immune reconstitution inflammatory syndrome (IRIS) with this treatment, an immune-induced inflammatory response to JCV that results in serious neuronal damage. We herein report a case of a 60-year-old female who suffered from PML 5 years after lung transplantation, had worsened brain lesions thought to be related to PML-IRIS at the time of immunosuppressant reduction, and missed treatment opportunities.

**Case presentation:**

A 60-year-old female developed PML 5 years after lung transplantation. Fluid-attenuated inversion recovery and diffusion-weighted brain magnetic resonance imaging (MRI) revealed multiple high-signal lesions, mainly in the cerebral white matter. Polymerase chain reaction found 0.32 million copies/mL of JCV in the cerebrospinal fluid. Thus, she was given a diagnosis of PML. Mycophenolate mofetil and tacrolimus dosages were reduced, and CD4-positive cell counts and the blood concentration of each immunosuppressant were monitored. Mefloquine was also orally administered at a daily dose of 275 mg for 3 days and was then administered at a dose of 275 mg per week. Although the patient’s CD4-positive cell counts increased and her immune system recovered, her symptoms and brain MRI findings worsened. We suspected PML progression or a transition to PML-IRIS. Steroid pulse therapy to suppress the inflammatory lesions was not possible but was retrospectively indicated. The patient rapidly began to exhibit akinetic mutism and died 4 months after the onset of neurologic symptoms.

**Conclusions:**

When neurologic symptoms and abnormal brain MRI findings are noted during immune recovery, it is often difficult to distinguish between progressed PML and PML-IRIS. However, the pathogenesis of brain lesions usually involves inflammation and immune-reactive mechanisms for JCV. Steroid pulse therapy, which can reduce inflammation, should thus be administered in organ transplantation cases with differential diagnoses including PML-IRIS.

## Background

Progressive multifocal leukoencephalopathy (PML) is a rare, progressive demyelinating disease of the brain, caused by reactivation of JC polyomavirus (JCV) in glial cells, especially in the white matter. A diagnosis of PML requires identification of JCV via polymerase chain reaction (PCR) in the cerebrospinal fluid (CSF) samples or via in situ *hybridization* of brain biopsy tissues [1]. PML is a critical and lethal CNS complication that often follows the kidney, liver, heart, lung, or bone marrow transplantation [2]. PML after lung transplantation is rarely reported, with only seven cases documented in the literature [2–7]. Treatment for PML is challenging and includes the use of mefloquine, cidofovir, and cytarabine, which inhibit JCV replication. Mirtazapine, a 5-HT2a receptor inhibitor that prevents the spread of JCV infections to oligodendrocytes, is also used in the treatment of PML [8]. In addition, the progression of PML may be interfered with immune recovery, particularly in cases where the use of immunosuppressants can be safely reduced or discontinued without causing organ rejection.

PML treatments are limited at present, and conventional therapies, such as mirtazapine, cidofovir, cytarabine, and mefloquine, are not effective and have side effects [8]. For instance, some treatments use passive and active immunization. Passive immunization utilizes recombinant human anti-JCV VP-1 monoclonal antibodies to neutralize JCV in the blood or central nervous system. A JCV-specific cytotoxic T lymphocyte may be generated by peripheral blood mononuclear cells from a stem cell donor, which may be stimulated with JCV VP-1 and large T antigens [9]. Therefore, innovative and useful treatments are urgently needed for PML.

Immune reconstitution inflammatory syndrome occurring after PML (PML-IRIS) is the inflammatory reaction that occurs near PML lesions when immunocompetence is recovered. Clinical symptoms and brain magnetic resonance imaging (MRI) findings often worsen in PML-IRIS. Recently, attempts have been made to differentiate PML-IRIS from progressed PML via serial gadolinium-enhanced MRI and T2-weighted images. However, it is still difficult to distinguish between these two diagnoses in many cases [10].

We herein present a case of a 60-year-old female who suffered from PML 5 years after lung transplantation, had aggravated brain lesions thought to be related to PML-IRIS at the time of immunosuppressive dose reduction, and missed the treatment opportunity.

## Case presentation

The patient was a 60-year-old female who visited our hospital because of progressive apathy. She was previously diagnosed with pulmonary lymphangioleiomyomatosis at 42 years of age and underwent a right lung transplant at 55 years of age. After transplantation, oral tacrolimus (1.9 mg), mycophenolate mofetil (MMF; 500 mg), and prednisolone (5 mg) were prescribed. She did not undergo post-operative home oxygen therapy and was able to perform housework without any problems. Three months before her hospitalization, the patient experienced dizziness, decreased motivation and activity, confabulations, and delusions. She received an influenza vaccination 6 weeks before her admission. Three weeks before her hospitalization, the patient began to make medication administration errors and experience urinary incontinence.

She was suspected by our hospital physician of having a neurological disorder and thus underwent brain MRI, and T2-weighted and fluid-attenuated inversion recovery (FLAIR) images revealed multiple, high-signal lesions in the white matter in the bilateral cerebral hemispheres. Based on these findings, the patient was admitted in our hospital.

Further examinations found that the patient was conscious but apathetic, with poor spontaneous speech. She had no cranial nerve abnormalities. Her tendon reflexes were generally exaggerated, and her Babinski reflex elicited plantar flexion without paralysis. The patient had no clear sensory impairments, cerebellar ataxia, autonomic nervous system disorders, or meningeal irritation. No abnormalities were noted in her physical examination.

Blood tests revealed leukopenia (2700/μL), anemia (9.0 g/dL), low CD4-positive lymphocytes (242/μL), hypo-γ-globulinemia (IgG 683 mg/dL), and mild renal dysfunction. Her thyroid function, glucose tolerance, and vitamin B1 and B12 levels were normal. The levels of tumor markers, including CA-19-9, CA 125, and CEA, were not elevated. No autoantibodies that could cause cerebrovasculitis were detected. Serum RPR, TPHA, anti-HBsAg, anti-HCV antibody, anti-HIV antibody, anti-HTLV-1 antibody, and interferon-gamma release assays such as the T-SPOT® were negative. CSF examinations, including cell counts, cell type, protein level, and glucose and chloride levels, were normal. β-D-glucan (< 6.0 pg/mL), sIL -2R (< 50 U/mL), myelin basic protein, and oligoclonal bands were all negative. In addition, CSF viral PCR and RT-PCR were negative for the varicella-zoster virus, herpes simplex virus types 1 and 2, human herpesviruses 6 and 7, cytomegalovirus, adenovirus DNA, and measles virus RNA. However, CSF PCR for JCV, performed at the National Institute of Infectious Diseases, was positive (0.32 million copies per mL).

**Fig. 1:**
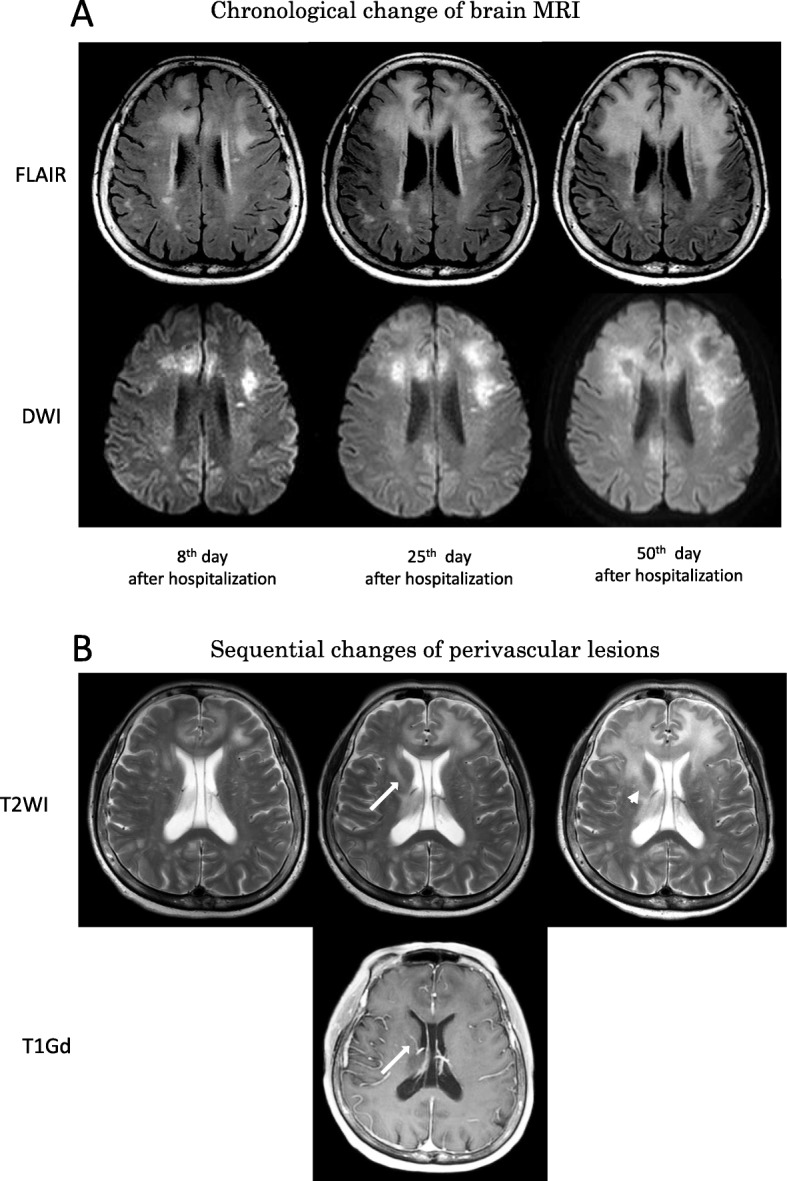
Imaging findings of this patient. **a** Chronological changes in brain magnetic resonance imaging (MRI), fluid attenuated inversion recovery (FLAIR; top row), and diffusion weighted imaging (DWI, bottom row) on an axial view. Eight days following the patient’s admission, a FLAIR image revealed a high signal lesion in the cerebral white matter, which was dispersed in the central bilateral frontal lobes. Signal elevations were also revealed on DWI. (left) Twenty-five days following the patient’s admission, the number of high signal white matter lesions increased on FLAIR and DWI, and the discrete lesions expanded. (middle) Fifty days following the patient’s admission, the lesion with an elevated FLAIR signal expanded further to cover the entire bilateral frontal lobes. On DWI, the patient’s lesions had altered signal facilitation in the center and a restricted signal along the edge with perilesional edema. (right). **b** T2-weighted magnetic resonance (MR) images (top row) demonstrated signal changes in the perivascular lesion. T1-weighted magnetic resonance imaging with contrast administration 25 days following the patient’s admission revealed enhanced vasculature (likely veins). (white arrow) Subsequently, a T2-weighted image taken 50 days after the patient’s admission revealed perivascular edema around the indicated blood vessel. (white arrowhead) Similar perivascular edema was identified in nearby blood vessels.

Brain MRI on the patient’s eighth day of hospitalization revealed high-intensity lesions on both T2-weighted and FLAIR imaging in the cerebral white matter in the bilateral frontal lobes. These lesions were enlarged relative to those observed on the patient’s initial MRI at admission. These same lesions appeared on high-intensity diffusion-weighted imaging (DWI) and low-intensity T1-weighted imaging (Fig. 1). No abnormal accumulation was detected using brain thallium scintigraphy. The patient’s electroencephalogram was within the normal range, and no epileptiform discharge was noted.

Because the patient had a compromised immune system owing to use of several immunosuppressant drugs following her lung transplantation, differential diagnoses were considered, including PML, viral encephalopathy, delayed graft versus host disease vasculitis, tacrolimus encephalopathy, and lymphoproliferative disease. Acute disseminated encephalomyelitis (ADEM) was also suspected due to the patient’s post-vaccination status.

**Fig. 2:**
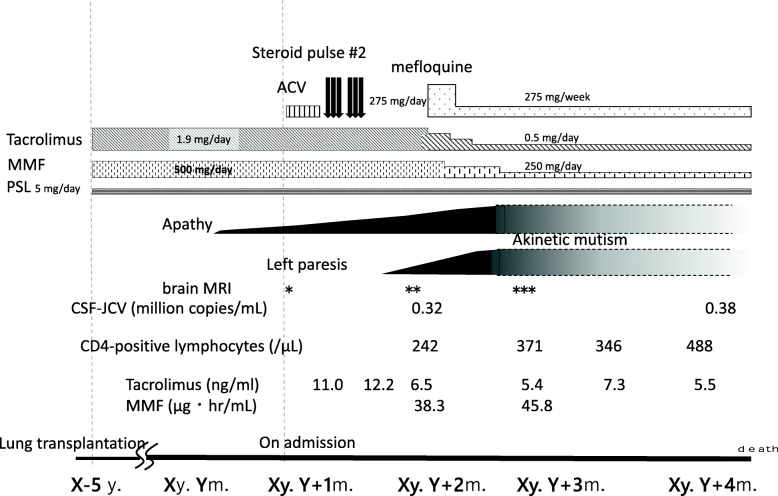
Clinical course. Mycophenolate mofetil (500 mg) and tacrolimus (1.9 mg) were administered after the patient was released from the hospital. The patient’s excess apathy was noted 5 years after her lung transplant. This patient was admitted in our hospital 1 month after onset of apathy. Herpes encephalitis and acute disseminated encephalomyelitis were suspected at first. Accordingly, acyclovir and steroid pulse therapies were administered but were ineffective. Furthermore, JC polyoma virus (JCV)-PCR revealed transcript concentrations of 0.32 million copies/mL in the cerebrospinal fluid. Given these findings and those from magnetic resonance imaging (MRI), the patient was diagnosed with progressive multifocal leukoencephalopathy. Immunosuppressant drug levels were reduced, and an acute rejection episode was monitored via CD4-positive cell counts. Mefloquine treatment was also started. Although this patient’s CD4-positive cell counts gradually increased, her clinical symptoms progressed. She exhibited akinetic mutism 1 month after hospitalization (2 months after onset of PML). Despite increased CD4-positive cell counts, JCV-PCR copy numbers in the cerebrospinal fluid did not decrease, and her symptoms did not improve. Three months after her initial hospitalization (4 months after symptom onset), the patient died due to PML-related complications *Brain MRI performed on admission; **Brain MRI performed 25 days after admission; ***Brain MRI performed 50 days after admission.

As shown in Fig. 2, because the result of HSV-PCR of the CSF sample was negative, administration of acyclovir was discontinued 5 days after the patient’s admission. Brain MRI performed 25 days after the patient’s admission confirmed the enlargement of white matter lesions in the bilateral frontal lobes and revealed a faint gadolinium contrast effect in the left frontal lesion. At this time, the patient’s CSF was found to be positive for JCV-DNA. Given her brain MRI findings, she was diagnosed with probable PML.

The patient’s tacrolimus and MMF immunosuppressant dosages were respectively reduced from 1.9 to 0.5 mg and from 500 to 250 mg in 5 weeks. At the same time, mefloquine (275 mg/day) was administered for 3 days as a therapeutic agent according to the Japanese PML practice guidelines. Its use had been reported previously for PML after lung transplantation [2, 5] (Table 1). Following immunosuppressant dosage reductions, the patient’s CD4-positive lymphocyte levels, an indicator of immunosuppression, gradually increased. However, brain MRI performed 50 days after the patient’s admission revealed that the patient’s lesions had expanded and swelled due to a mass effect [10]. The enlarged lesion had a low central signal intensity on DWI but a high signal intensity along its periphery (Fig. 1) [11, 12]. Although the patient’s CD4-positive cell counts further recovered 81 days after the patient’s admission, CSF JCV levels increased to 0.38 million copies/mL. The patient regrettably died due to PML-related complications 91 days after her admission.

**Table 1:** Clinical features of the past reported cases that developed PML after lung transplantation

Case (year)	Age /Sex	Immuno- Suppressant regimen	Time to PML onset from transplantation (Mo)	Clinical Symptoms	Method of diagnosis (specimen)	Reduced immunosupressant as therapy	Treatment for PML	From PML onset to diagnosis (Mo)	Outcome (cause of death/ period from onset to death; Mo)
Ouwens JP (2000) [3]	43/M	AZA CsA CS	15	Visual loss, seizure, visual hallucination, ataxia, rt.paresis, dysarthria, memory impairment	PCR (CSF)	RD Change (AZA→MMF→CsA)	-	13	Died (PML / 15)
Waggoner J (2009) [4]	38/F	AZA,Tac, CS,Alemtuzumab,	43	Gait instability (ataxia) Visual changes, confusion	PCR (CSF)	RD	cidofovir mirtazapine	1.5 ~ 2	Died (respiratory failure / 5)
Mateen FJ (2011) [2]	39/F	-	42	Ataxia	PCR (CSF)	RD	mirtazapine, mefloquine	3	Died (ND / 9.1)
Mateen FJ (2011) [2]	62/F	-	27	Memory impairment, Ataxia, lt. hemiparesis	PCR (CSF)	RD	-	< 1	Died (ND /15.6)
Lobo LJ (2013) [5]	61/M	Rituximab CsA MMF CS	13	Headache, Malaise Memory impairment	PCR (CSF)	RD	-	ND	Neurologic symptoms exacerbation, move to hospice
Moua T (2013) [6]	61/M	Tac CS	5	Rt. Hemiparesis & sensory disturbance, cognitive decline, aphasia	ISH (brain biopsy)	RD	cytarabine	3	Died (ARE?/ 7)
Panchabhai TS (2016) [7]	60/F	Tac CS	16	Lt. arm paresis, Memory impairment, visual deficits, emotional lability,	PCR (BALF & CSF)	Change (Tac→rapamycin), Discontinued	-	2	Died (respiratory failure: ARE / 3)
Present case	60/F	Tac MMF CS	61	Apathy	PCR (CSF)	RD	mefloquine	2	Died (respiratory failure / 5)

*AZA* Azathioprine, *CsA* Cyclosporine, *CS* Corticosteroid, *Tac* Tacrolimus, *MMF* Mycophenolate mofetil, *CSF* Cerebrospinal fluid, *PCR* Polymerase chain reaction, *ISH* In-situ hybridization, *BALF* Bronchoalveolar lavage fluid, *RD* Reduced doses, *ND* Not described, *ARE* Acute rejection episode,

## Discussion and conclusions

To date, seven cases of PML that occurred following lung transplantation have been reported in the literature [2–7]. In these cases, different immunosuppressive drugs were used at PML onset, and more than two immunosuppressants were administered in each case. Some cases occurred after intensive immunosuppression therapy or administration of alemtuzumab, rituximab, or steroid pulse therapy, which was used due to graft rejection. Tacrolimus was used in the present case, as well as in three out of the seven cases. Up to the present, no studies have suggested a relationship between tacrolimus use and PML.

Notably, the patient in the study by Shitrit et al. was diagnosed with PML following brain biopsy, and the causative virus was BK virus rather than JCV. The effectiveness of cidofovir and its therapeutic reactivity can be different in PML with the presence of JCV [13]. We thus eliminated this case from our literature review (Table 1). The literature review included one case of mefloquine and mirtazapine use, [2] one case of cidofovir and mirtazapine use, [4] one case of cytarabine alone, [6] and three untreated cases [2, 3, 7] of PML after lung transplantation. (Table 1) Because cases of PML after organ transplantation are rare, this literature review provides a significant summary of published cases of PML after organ transplantation.

Several studies showed that decreasing immunosuppressant usage led to preferable outcomes. In fact, in the included studies, all survivors of PML reduced or discontinued immunosuppressant use to restore their immunity against JCV, regardless of whether anti-JCV therapy was administered [14–18]. Of the seven post-lung-transplantation PML patients, five had immunosuppressant reductions or discontinuation [2, 4–6], and two underwent treatment with other immunosuppressants (Table 1) [3, 7]. However, few reports have identified useful indicators for immunosuppressant reductions [4].

Accumulating evidence indicates that CD4 is not an indicator of PML, and PML can develop at any CD4 level, although low CD4 cell counts are often associated with the risk of PML. In a case investigated by Waggoner et al., PML developed 1 year after treatment with alemtuzumab (CD4-positive cell count: 41/μL). Despite a decreased immunosuppressant dose, this patient’s CD4-positive cell count increased to 162/μL, indicating PML progression [4]. Conversely, in another case of PML after liver transplantation, discontinuation of an immunosuppressant led to increased CD4-positive cells (648/μL), and with mefloquine treatment and immunosuppressant reduction, both lesion expansion and neurologic symptom progression halted [19]. Therefore, increased CD4 cell counts may serve as a marker of immune reconstitution.

PML-IRIS is characterized by an exacerbation of neurologic symptoms despite partial or full immune recovery and previously immunocompromised status. Clinically differentiating between PML and PML-IRIS is often difficult. Attempts have been made to distinguish between these conditions using sequential differences in brain MRI findings, and further identification of different inflammatory patterns using MRI is expected [10]. Brain MRI findings, including new swelling with perilesional and perivascular edema, are often consistent in PML-IRIS cases. Sequential gadolinium enhancement MRI may be used in some cases to further identify imaging abnormalities, although this approach was not used in the present case because of the patient’s pre-existing renal dysfunction. Most importantly, despite the recovery of CD4 positive cell counts, neurological symptoms and brain MRI findings were exacerbated in the present case. Therefore, a diagnosis of PML-IRIS was strongly suspected upon retrospective consideration. If steroid pulse therapy had been administered at the time of symptom onset or upon discovery of worsening brain MRI findings, this patient might have survived longer. Critically, JCV levels may not necessarily reflect the progression of PML in the advanced stage [20].

Active immunization has been proposed for the treatment of PML, and the JCV vaccine can be used to prevent PML development by boosting JCV VP1 antigen levels using recombinant interleukin-7 [21] or JCV peptide antigens inoculated via the intestinal tract [8]. Recently, pembrolizumab or nivolumab, which block PD-1 suppressing JCV clearance, have been demonstrated to effectively treat PML [22, 23]. In addition, injection of allogeneic BK virus-specific T cells may also be effective for treating PML and controlling IRIS [24]. However, these treatments have limited efficacy and severe side effects. Therefore, further studies are needed to develop highly safe therapeutic agents that directly inactivate JCV.

In conclusion, PML after lung transplantation under the conditions described above has a poor prognosis. PML treatment must allow the patient’s immune system to recover sufficiently so that he/she can combat PML without sacrificing organs required for life. Distinguishing IRIS from PML may be difficult; however, once immune reconstitution is underway, treatment for suspected IRIS, which has been demonstrated to be beneficial in some circumstances, should be administered when the conditions are similar to those in the present case.

## Data Availability

Data generated during this study are included in this published article.

## Abbreviations

ADEM: Acute disseminated encephalomyelitis
DWI: Diffusion weighted image
FLAIR: Fluid-attenuated inversion recovery
IRIS: Immune reconstitution inflammatory syndrome
JCV: JC polyoma virus
MMF: Mycophenolate mofetil
MRI: Magnetic resonance imaging
PCR: Polymerase chain reaction
PML: Progressive multifocal leukoencephalopathy
Tac: Tacrolimus
